# A delayed diagnosis of iatrogenic ureteral injury results in increased morbidity

**DOI:** 10.1038/s41598-024-63847-6

**Published:** 2024-06-14

**Authors:** Rachel Maheswaran, Christian Beisland, Anne K. Bergesen, Bjarte Almås

**Affiliations:** 1 Department of Urology, Haukeland University Hospital, 5021 Bergen, Norway; 2 Department of Clinical Medicine, University of Bergen, Bergen, Norway

**Keywords:** Ureter, Epidemiology, Risk factors

## Abstract

This study aimed to register and analyse outcomes after iatrogenic ureteral injuries (IUI) with special emphasis on potential consequences of a delayed diagnosis, and further to analyse if the incidence of IUI has changed during the study period. 108 patients treated for an IUI during 2001–2021 were included. Injuries due to endourological procedures, planned tumour resection and traumatic injuries were excluded. All relevant information to answer the research questions were entered into a database. Chi-square and t-tests were used for categorical and continuous variables respectively. Regression analysis was used to evaluate potential change of incidence in IUIs over time. Our results showed that most IUIs (74, 69%) were caused by gynaecological surgery. 49 (45%) had a delayed diagnosis (not diagnosed intraoperatively). Younger age (mean 50 vs 62 years, p < 0.001) and benign indication for laparoscopic hysterectomy (OR 8.0, p < 0.001) predisposed for a delayed diagnosis. Patients with a delayed diagnosis had a higher number of secondary injury related procedures (mean 4.6 vs 1.7, p < 0.001), hospital admissions (mean 3.0 vs 0.8, p < 0.001) and longer hospital stays (mean 20.6 vs 3.9 days p < 0.001) compared to patients with an intraoperative diagnosis. There was complete recovery for 91% of the patients. We did not observe any changes in IUI incidence during the study period. In conclusion, our study underlines that IUI can cause major morbidity for the patient affected if not diagnosed intraoperatively. Benign indication and younger age are predictors for a delayed diagnosis. The prognosis is good, with 91% full recovery. No significant changes in incidence of IUIs were observed.

## Introduction

Iatrogenic ureteral injury (IUI) can cause significant morbidity and potential detrimental long-term consequences for the patient affected. They most frequently occur to the distal part of the ureter, and the most common causes are endourological injuries due to kidney stone removal and laparoscopic gynaecological and gastrointestinal surgery both due to benign and malignant diagnoses^1–3^.

Previous publications indicate potentially severe consequences of IUIs with a delayed diagnosis, compared to injuries diagnosed intraoperatively. Patients with a delayed diagnosis may sustain severe morbidities needing hospital readmissions and several secondary medical procedures awaiting definite treatment, and some may also experience life threatening complications due to the injury^4–6^. There is also the matter of increased costs to the healthcare system related to IUIs. In a Norwegian study on IUIs during laparoscopic hysterectomies, 11% (69 of 634) applied for patient injury compensation, whereof 15% were granted. In the same study, 17% suffered long-term consequences such as nephrectomy or chronic pain^7^.

The purpose of our study was to register IUIs treated at our tertiary referral centre during 2001–2021, and to assess the morbidity following a delayed diagnosis. Furthermore, we wanted to investigate potential changes over time regarding the incidence of IUIs.

## Material and methods

### Patient selection

The study was approved as a clinical audit after being evaluated by the Regional Ethics Committee for scientific research in Norway (ref. number 543539–22). Registered diagnostic codes (ICD-10) and procedural codes (NMCP) registered at the hospital’s local operation planner (Orbit^©^ version 4 and 5) were used to identify potential candidates for inclusion. We included all patients surgically treated for iatrogenic ureteral injuries at Haukeland University Hospital (HUH) from 2001 through 2021. We consider IUIs sustained during endourological procedures to be a separate entity regarding aetiology and treatment, and these patients were therefore excluded from the study. We also excluded traumatic injuries (n = 2) and intentional injuries necessary to secure radical tumour resection. The electronic medical records were reviewed, and information about age, gender, former laparotomies, type of surgery performed when the injury occurred, degree of injury, time until diagnosis, temporary treatment, and number of procedures related to the injury were registered. We also recorded the number of hospital admittances, total number of days admitted, definite treatment and final outcomes (control after 1 year) including kidney function. We entered all relevant data to the database.

### Primary aim: consequences of a delayed diagnosis

To address our primary aim, we divided the cohort into two groups. One group with patients whose injuries were diagnosed intraoperatively, and one group with patients diagnosed after the main procedure and thus considered with a delayed diagnosis. The groups were compared regarding time to diagnosis, number of procedures performed, number of hospital admissions, total length of hospital stay, time until definite treatment and final outcomes including the effect on kidney function.

### Secondary aim: changes in incidence over time

To address possible changes in incidence over time, we analysed the number of injuries per year during the study period. To assess potential changes in the IUI incidence relative to the total number of procedures, we used hysterectomies performed at our local gynaecology department from 2007 through 2021 as a reference. This because hysterectomies were a common cause for IUIs, and complete data on the total number of hysterectomies performed during the time-period was available.

### Statistical analysis

We used IBM SPSS statistics version 26^©^ for statistical analyses. Chi-square and t-tests were performed to assess predictors for a delayed diagnosis and post injury morbidity. Linear regression was used to analyse potential changes in IUI incidence over time. p-values less than 0.05 were considered statistically significant.

### Ethics approval

The study was approved as a clinical audit, and the need for informed consent was waived by the Regional Ethics Committee for scientific research in Norway (ref. number 543539-22). The study was conducted in order with relevant guidelines and regulations.

## Results

We included 108 patients who sustained an IUI during the period 2001 through 2021. Characteristics of the patients and type of surgery are summarised in Table 1.

**Table 1:** Patient and injury characteristics, and description of surgery performed during injury.

Patient characteristics	n = 108 (100%)
Gender	
Men	24 (22%)
Women	84 (78%)
Age (median)	56 years
Former laparotomy	
Yes	28 (26%)
No	80 (74%)
Anatomical location	
Proximal ureter	5 (5%)
Middle ureter	7 (6%)
Distal ureter	93 (86%)
Missing	3 (3%)
Degree of injury	
Laceration/perforation/ligation/unknown	62 (57%)
Total rupture	46 (43%)
Side	
Left	52 (48%)
Right	50 (46%)
Bilateral	6 (6%)
Type of surgery	
Laparotomy	78 (72%)
Laparoscopic or robot assisted surgery	30 (28%)
Injury by department	
Gynaecological	74 (69%)
Urologic	9 (8%)
Other^a^	25 (23%)
Procedure	
Hysterectomy	52 (48%)
Bowel resection	23 (21%)
Other	33 (31%)_1_
Time of diagnosis	
Intraoperatively	59 (55%)
Within 1 week	17 (16%)
Later then 1 week	32 (29%)

^a^Mainly gynaecological and gastrointestinal surgery, but also vascular and neurosurgery.

The patients with a delayed diagnosis were significantly younger than the patients who had their injuries diagnosed intraoperatively (mean 50 vs 62 years, p < 0.001). Among the IUIs, 52 (48%) occurred during a hysterectomy, of which 24 (46%) had a benign indication. Women with a benign indication for hysterectomy tended to be younger (mean 50 vs 54 years, p = 0.07), and were much more likely to have a delayed diagnosis (79% vs 32%, OR 8.0 CI 2.3–28, p < 0.001). Patients who underwent laparoscopic or robot-assisted surgery (n = 30) had a non-significantly higher risk for a delayed diagnosis compared to patients who underwent open surgery (63% vs 40%, p = 0.06).

All the patients diagnosed intraoperative received either temporary or definite treatment during the primary procedure. The median (IQR) time from injury to recognition for the patients with a delayed diagnosis was 11 (5–28) days. Median (IQR) days from injury to definite treatment for the same group was 109 (59–163) days. The patients with a delayed diagnosis had a significantly higher number of hospital admissions than the patients diagnosed intraoperatively (mean 3.0 vs 0.8, p < 0.001), and longer hospital stays (mean 20.6 days vs 3.9 days, p < 0.001). They also had a significantly higher number of procedures linked to the temporary and definite treatment for the injury (mean 4.6 vs 1.7, p < 0.001).

Most of the patients with a delayed diagnosis received temporary interventions awaiting definite treatment as described in Table 2. Of the patients, 6 (12%) patients did not receive any temporary treatment, mostly due to definite treatment being planned shortly after the time of injury.

**Table 2:** Temporary treatment for the patients with a delayed diagnosis.

Temporary treatment	n = 43 (100%)
Nephrostomy catheter	32 (74%)
Ureteral (JJ) stent alone	9 (21%)
Both nephrostomy and ureteral (JJ) stent	2 (5%)

The intended definite treatment for our patients is described in Table 3. Most were treated with ureteroneocystostomy (reimplantation to the bladder, n = 56) or primary anastomosis with or without a JJ-stent (n = 36). The patients who had IUIs treated with primary suture without JJ-stent were diagnosed intraoperative, and their injuries were described as ligation/perforation without total avulsion. Six were treated with a stent alone, while seven required more extensive surgery (ileal conduit urinary diversion n = 3, uretero-uretero crossover n = 2, nephrectomy n = 1 and renal autotransplant n = 1). The patients treated with ileal conduit urinary diversion were all male with a history of rectosigmoid cancer where the injury occurred during primary cancer surgery, or surgery due to cancer recurrence. They all had a delayed diagnosis of IUI. The degree of IUI, former surgery and the recurrence of cancer affected the surgeons’ choice of choosing ileal conduit in these three cases. One very comorbid patient was treated with permanent drainage with a nephrostomy catheter alone.

**Table 3:** Definite treatment for all the patients in the study group.

Definite treatment	n = 108 (100%)
Ureteroneocystostomy	56 (52%)
Primary suture and (JJ) stent	36 (33%)
Ureteral (JJ) stent alone	6 (5%)
Primary suture without ureteral (JJ) stent	2 (2%)
Nephrectomy	1 (1%)
Uretero-uretero crossover	2 (2%)
Ileal conduit urinary diversion	3 (3%)
Renal autotransplant	1 (1%)
No treatment	1 (1%)

### Outcome

Data regarding outcome could be retrieved for 91 of our 108 patients, as follow-up sometimes were organised in other regions not covered by our medical records or that the patient was lost to follow-up*.* We defined eight (9%) patients to have suffered a sequela. Among these one needed a nephrectomy, three received permanent drainage with nephrostomy or ureteral (JJ) stent, three received an ileal conduit urinary deviation, and one went through a renal autotransplantation. None of the patients developed fistula as a sequalae after the IUI. 83 (91%) of the patients did not have a sequela one year after definite treatment, despite 45% of them having a delayed diagnosis. Of the patients who suffered permanent sequelae (9%), four were diagnosed intraoperatively and four were diagnosed late. Thus, we did not find that time to diagnosis was related to outcome. There were no deaths related to the IUIs.

Finally, we compared kidney function by use of serum creatinine before the procedure and one year after. Only two patients developed kidney failure after IUIs in our material, and none of these could be directly related to the injury.

### Secondary aims

**Figure 1:**
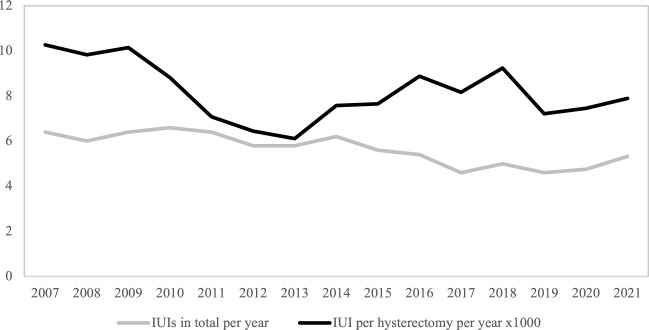
Line graph depicting incidence of IUIs during hysterectomies in total per year, and per hysterectomy per year, from 2007 to 2021.

We have complete data on hysterectomies performed at the gynaecological department HUH during 2007–2021. There were 4925 hysterectomies performed in this time-period. Of these, 40 (0.8%) sustained an IUI. Development of IUIs over time is illustrated in Fig. 1. There was a non-significantly increase in the number of IUIs treated at our hospital per year, with a yearly increase of 0.11 (CI − 0.06 to 0.29, p = 0.2). The proportion of IUIs per hysterectomy decreased non-significantly during the study period, with an estimated yearly decrease of 0.011% (CI − 0.07 to 0.05%, p = 0.7). To summarize, we found no significant changes regarding the number of injuries treated, or incidence relative to hysterectomies performed, during the study period.

## Discussion

The main finding of the present paper was that a delayed diagnosis of an IUI imposes severe negative consequences for the affected patients. Patients with a delayed diagnosis experienced a higher number of procedures, hospital admissions, and longer hospital stays compared to those diagnosed intraoperatively.

In our material 45% of the patients had a delayed diagnosis. Even though most of the patients with a delayed diagnosis had a complete recovery, these patients were in excess young and middle-aged women who went through several procedures and hospital readmissions, afflicting their lives in a grave manner. Procedures were for example inserting and changing of nephrostomy catheters and ureteral stents. In our cohort, 69% of the patients with a delayed diagnosis needed a nephrostomy catheter as temporary treatment for a prolonged period. The hospital readmissions were mostly due to following procedures or due to infection requiring intravenous antibiotics. This is supported by Blackwell et al.^4^, who found increased risk for sepsis and urinary fistula for patients diagnosed late after IUIs during hysterectomies. Consequently, our patients diagnosed late had several days admitted to the hospital, and thus longer hospital stays. The median (IQR) time from injury to final treatment was 109 (59–163) days. The waiting time were for some patients dominated by infection and pain, and probably sick leave even though we do not have data on this. This is similar to what Locke et al.^5^ found in their retrospective study, even though they had fewer injuries with a delayed diagnosis (21 of 103, 20%) than in our material. In addition, they found that laparoscopic surgery had a higher risk of delayed diagnosis compared to open approach. They did not find any other predictors regarding previous surgery, previous radiation, diagnosis, type of surgery or demographics. Wei et al.^8^ found that surgeon inexperience was the main prime risk for these injuries, and Ali et al.^6^ found that late diagnosis of IUIs were more common amongst non-specialist surgeons.

We found a non-significantly increased risk of delayed diagnosis when the IUI occurred during laparoscopic or robot-assisted surgery. Several meta-analyses have found higher incidence for iatrogenic IUI during laparoscopic hysterectomies, compared to hysterectomies with open approach^9,10^. Parpala-Spårman et al.^11^ found that there was a higher risk for delayed diagnosis during gynaecological laparoscopic surgery, correlating with our material. They also found an increasing incidence of IUIs between 2000 and 2006. Al-Awadi et al.^3^ found a decreasing incidence of IUIs the last years attributed to prophylactic stenting prior to major abdominopelvic surgery. Both prophylactic stenting and cystoscopy looking for ureter efflux have been discussed being used as preventive measures to avoid a delayed diagnosis or to prevent IUIs altogether^12–15^. Prophylactic stenting is though still controversial due to possible side effects as infection and acute renal failure in addition to increased costs to the health care system^16,17^.

Patients having a benign indication for hysterectomy were more likely to have a delayed diagnosis compared to a malignant. Of all the hysterectomies, 46% were due to a benign diagnosis. Ravlo et al.^7^ found that patients sustaining IUI during hysterectomy on benign indications were more likely to apply for patient injury compensation. Diagnosis after discharge and younger age were also associated with application for injury compensation.

The reason why younger age and benign indication for hysterectomy predispose for a delayed diagnosis is not clear to us, but one theory that already has been mentioned is surgeon experience^6,8^. Our theory is that the more experienced surgeons perform surgery on malignant indication, due to the importance of radical tumour resection. Less experienced surgeons may operate on younger, less comorbid patients with benign indication where radical tumour resection is irrelevant, and therefore be less attentive to IUI. Regrettably, we do not have data on surgeon experience in our database.

Even though patients with a delayed diagnosis may suffer through infections, interventions, hospital readmissions and longer hospital stays, the prognosis is good. This seems to be the findings in other publications as well^18,19^. It is important for surgeons performing abdominal surgery to be aware of this to decrease the risk of morbidity for their patients.

The study has strength in our relatively large patient database including patients followed for two decades at our tertiary referral centre, giving us representative data on the injuries, treatment, and outcome. However, it is a retrospective study, and correct documentation and interpretation of the data available in the medical journals is crucial. In addition, seventeen of the patient's follow-up data are missing due to patients lost to follow-up, which may affect our outcome.

## Conclusion

The consequences of an IUI with a delayed diagnosis can be severe for the patient affected, and surgeons performing abdominal surgery should therefore be watchful. Benign indication and younger age predispose for a delayed diagnosis. The prognosis is good. There does not seem to be a change in the incidence of IUI over time at our tertiary referral centre.

## Data Availability

The datasets generated and analysed during the current study are available from the corresponding author on reasonable request.
